# Egg parasitoid exploitation of plant volatiles induced by single or concurrent attack of a zoophytophagous predator and an invasive phytophagous pest

**DOI:** 10.1038/s41598-019-55396-0

**Published:** 2019-12-12

**Authors:** Letizia Martorana, Jacques Brodeur, Maria Cristina Foti, Alfonso Agrò, Stefano Colazza, Ezio Peri

**Affiliations:** 1 University of Palermo, Department of Agricultural, Food and Forest Sciences, Viale delle Scienze, 90128 Palermo, Italy; 2 Institut de Recherche en Biologie Végétale – Université de Montréal, Québec, Canada

**Keywords:** Agroecology, Invasive species

## Abstract

Zoophytophagous insect predators can induce physiological responses in plants by activating defence signalling pathways, but whether plants can respond to facultative phytophagy by recruiting natural enemies remains to be investigated. In Y-tube olfactometer bioassays, using a system including a *Vicia faba* plant, the zoophytophagous predator *Podisus maculiventris* and the egg parasitoid *Telenomus podisi*, we first demonstrated that *T. podisi* females are attracted by broad bean plants damaged by feeding activity of *P. maculiventris* and on which host egg masses had been laid, while they are not attracted by undamaged plants or plants damaged by feeding activity alone. In a second experiment, we evaluated the impact of the invasive phytophagous pest *Halyomorpha halys* on this plant volatile-mediated tritrophic communication. Results showed that the invasive herbivorous adults do not induce plants to recruit the native egg parasitoid, but they can disrupt the local infochemical network. In fact, *T. podisi* females are not attracted by volatiles emitted by plants damaged by *H. halys* feeding alone or combined with oviposition activity, nor are they attracted by plants concurrently infested by *P. maculiventris* and *H. halys*, indicating the specificity in the parasitoid response and the ability of the invasive herbivore in interrupting the semiochemical communication between plants and native egg parasitoids. To the best of our knowledge, this is the first study showing that zoophytophagous predator attacks induce indirect plant defences similarly to those defence strategies adopted by plants as a consequence of single or concurrent infestations of herbivorous insects.

## Introduction

Phytophagous insects are known to induce changes in the volatile emission profile in the plants they attack^1,2^. In response to phytophagous insect activities, plants emit volatile organic compounds (VOCs) that act as an indirect plant defence since they can recruit parasitoids of the herbivores^3^. These plant synomones are called herbivore-induced plant volatiles (HIPVs) or oviposition-induced plant volatiles (OIPVs), if they are emitted by the plants as a consequence of insect feeding damage or insect oviposition activity, respectively^4,5^. The exploitation of OIPVs is one of the main strategies adopted by egg parasitoids to optimize their foraging behaviour^6–12^. Indeed, OIPVs facilitate host egg location as they are easily detectable, being produced in large quantities by the plants, and are reliable indicators of the presence of host eggs^13,14^.

As is the case with solely phytophagous insects, plants can also provide supplemental food to omnivorous insect predators that feed on plant resources when prey become scarce^15,16^. However, this strategy of feeding at more than one trophic level not only has ecological consequences, but also might affect biological control programmes^17^. In fact, zoophytophagous predators, also called plant-feeding predators or facultative predators^18^, can be efficient natural enemies of phytophagous pests but also can damage plant tissues and, as a consequence, may inflict economic losses by feeding on crop plants when prey become scarce^17,19,20^. For example, the mirid bug *Nesidiocoris tenuis* (Reuter) is a useful control agent of several tomato pest (e.g. whiteflies, thrips, leafminers, aphids, mites, lepidopterans) but occasionally, its feeding activity can damage the plants^21^. Nevertheless, it was demonstrated that the feeding and/or oviposition activities of zoophytophagous species can activate host plants defence mechanisms that affect performance of other insect herbivores and their natural enemies^22–24^. In fact, the feeding activity of *N. tenuis* on tomato plants activates both direct and indirect plant defences by reducing the infestation of the pest whitefly *Bemisia tabaci* (Gennadius) and by attracting its parasitoid, *Encarsia formosa* (Gahan)^25–27^.

The spined soldier bug, *Podisus maculiventris* (Say) (Heteroptera: Pentatomidae), is a commercialized zoophytophagous predator of several agricultural and forest pests^28,29^, but when preys are scarce can feed on plants without causing crop injury^30^. *Telenomus podisi* (Ashmead) (Hymenoptera: Scelionidae), is an egg parasitoid of various phytophagous pentatomids, including the spined soldier bug^31^. This wasp can exploit VOCs emitted by plants on which pentatomid bugs feed to locate its associated hosts^32,33^. In this study we hypothesized that *Vicia faba* L. plants attacked by *P. maculiventris* emit OIPVs that recruit the associated egg parasitoid *T. podisi*. Therefore, we performed a series of bioassays to evaluate the response of *T. podisi* to volatiles induced in plants damaged by the zoophytophagous predator and associated host in order to characterize the local infochemical web.

Plants in the field are normally exposed to various herbivorous insects acting simultaneously or sequentially^34,35^. Recent literature, based on study systems comprised of concurrent plant-attacker combinations, showed that OIPVs can change and sometimes disrupt egg parasitoid recruitment^36,37^. For example, concurrent plant infestation by above- and below-ground herbivores disrupts the attraction of an egg parasitoid to OIPVs emitted by bean plants infested with a stinkbug pest^38^. Furthermore, species invasion can alter local trophic interactions toward various outcomes by adding new resources or by introducing novel interactions into local communities^39–41^.

**Figure 1:**
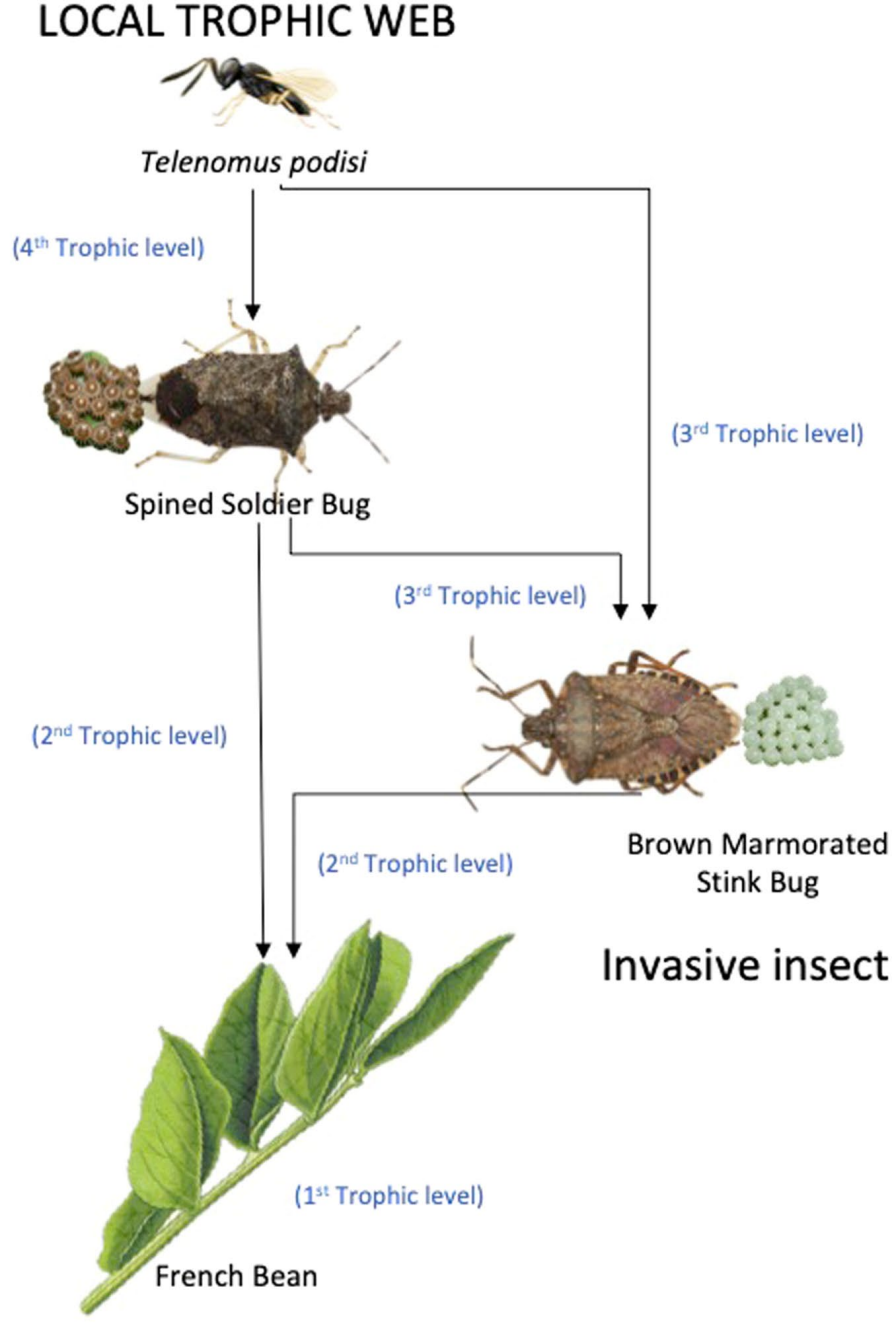
Schematic diagram of the studied multitrophic system. The arrows refer how species relate to each other.

The brown marmorated stink bug, *Halyomorpha halys* (Stål) (Heteroptera: Pentatomidae), is a pest of Asian origin that has recently spread in several European and North American countries, becoming a very common and destructive pest in orchards and field crops^42,43^. In Europe, it was shown that *H. halys* can disrupt the local tritrophic system *V. faba* - *Nezara viridula* (L.) - *Trissolcus basalis* (Wollaston)^44^. In fact, *T. basalis* females, which usually are attracted to OIPVs emitted by plants infested by *N. viridula*^45,46^, do not respond to plants on which *H. halys* has fed and oviposited alone or concurrently with the local pentatomid^44^. In North America, *H. halys* can directly interact with the local tritrophic system consisting of *V. faba*, *P. maculiventris* and *T. podisi* as shown in Fig. 1. In particular, *H. halys* attacks *V. faba* plants and it inhabits the same habitats as *P. maculiventris*^47^. *Podisus maculiventris* can feed on several pentatomid pests including *H. halys* eggs^48,49^. Finally, *H. halys* eggs are parasitized by *T. podisi*^50^, although if they are unsuitable hosts for egg parasitoid offspring development, therefore representing an evolutionary trap^51^. In this scenario, we hypothesized that the establishing of *H. halys* affects the chemical communications in the local tritrophic systems. Specifically, we evaluated the response of *T. podisi* to volatiles induced by plants infested by the invasive pest and non-associated host *H. halys*, in order to assess the ability of a local egg parasitoid to exploit HIPVs and initiate the host selection behavioural sequence towards an invasive stink bug. We next evaluated the response of *T. podisi* to volatiles emitted by plants subjected to concurrent infestation of *P. maculiventris and H. halys*, in order to address how the invasive *H. halys* could shape volatile-mediated signalling in a local tritrophic web and impact the structured insect communities.

## Results

All 35 tested *T. podisi* females responded to volatiles, and were included in the analyses.

**Figure 2:**
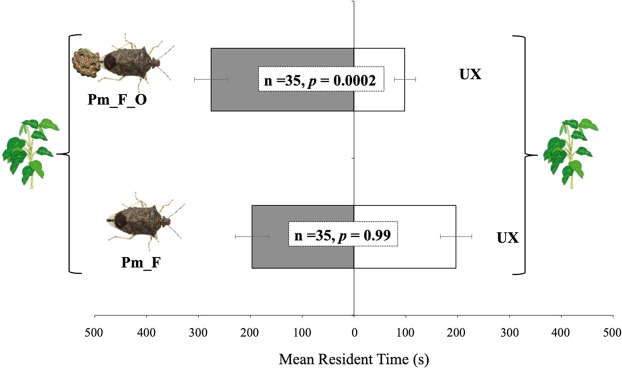
Response of *Telenomus podisi* females to *Vicia faba* plant volatiles induced by *Podisus maculiventris*. Plant treatments: *P. maculiventris* feeding and oviposition (Pm_F_O); *P. maculiventris* feeding (Pm_F); unexposed (UX). n = number of replicates. Bars represent mean (±SE) of the time spent by female wasps in each arm of a Y-tube olfactometer over an observation period of 600 sec (paired t-tests).

The response of *T. podisi* to plant volatiles induced by *P. maculiventris* activity is shown in Fig. 2. Wasp females showed a significant preference for broad bean plants damaged by feeding and oviposition activity of *P. maculiventris* over the unexposed plants (t = 4.12; df = 34; *p* = 0.0002). In contrast, there was no significant difference in preference to volatiles from bean plants damaged by *P. maculiventris* feeding or volatiles from unexposed plants (t = −0.006; df = 34; *p* = 0.99).

**Figure 3:**
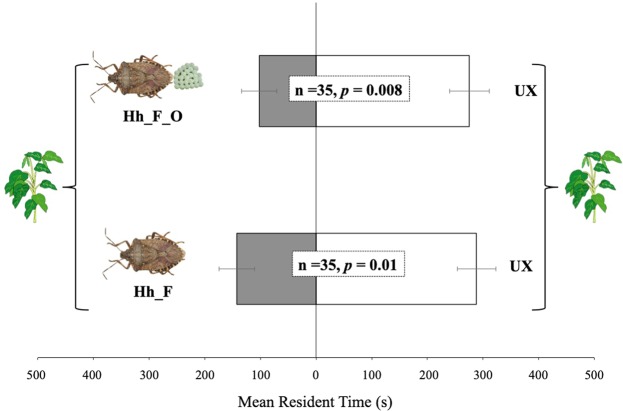
Response of *Telenomus podisi* females to *Vicia faba* plant volatiles induced by *Halyomorpha halys*. Plant treatments: *H. halys* feeding and oviposition (Hh_F_O); *H. halys* feeding (Hh_F); unexposed (UX). n = number of replicates. Bars represent mean (±SE) of the time spent by female wasps in each arm of the Y-tube olfactometer over an observation period of 600 sec (paired t-tests).

The response of *T. podisi* to plant volatiles induced by *H. halys* and non-associated host is reported in Fig. 3. Wasp females were not attracted to *V. faba* plants infested by *H. halys* relative to the unexposed plants. Indeed, unexposed plants were significantly more attractive to *T. podisi* than plants damaged by *H. halys* feeding and oviposition activities (t = −2.8; df = 34; *p* = 0.008) or by *H. halys* feeding (t = −2.47; df = 34; *p* = 0.018).

**Figure 4:**
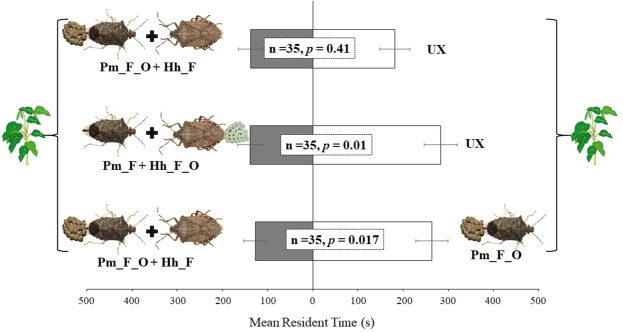
Response of *Telenomus podisi* females to *Vicia faba* plant volatiles induced by concurrent infestation of *Halyomorpha halys* and *Podisus maciliventris*. Plant treatments: *P. maculiventris* feeding and oviposition (Pm_F_O); *P. maculiventris* feeding and oviposition and *H. halys* feeding (Pm_F_O + Hh_F); *P. maculiventris* feeding and *H. halys* feeding and oviposition (Pm_F + Hh_F_O); unexposed (UX). Bars represent mean (±SE) of the time spent by female wasps in each arm of the Y-tube olfactometer over an observation period of 600 sec (paired t-tests).

The response of *T. podisi* to plant volatiles emitted by plants subjected to concurrent infestation of *P. maculiventris* and *H. halys* is shown in Fig. 4. Wasps did not prefer plants that were concurrently exposed to *P. maculiventris* feeding and oviposition activity and *H. halys* feeding, over unexposed plants (t = −0.83; df = 34; *p* = 0.41). When volatiles from plants damaged by *P. maculiventris* feeding and *H. halys* feeding and oviposition were tested against unexposed plants, *T. podisi* females were attracted by the latter (t = −2.62; df = 34; *p* = 0.01). Female wasps exhibited a significant preference for volatiles released by plants damaged by *P. maculiventris* feeding and oviposition when tested *vs*. plants concurrently damaged by *P. maculiventris* and *H. halys* (t = −2.48; df = 34; *p* = 0.017).

## Discussion

In this paper, in the first set of experiments, we show that the zoophytophagous predator *P. maculiventris* induces the host plant to emit OIPVs that recruit its associated egg parasitoid, *T. podisi*. This finding indicates that the oviposition of a pentatomid predator with facultative phytophagy can induce the host plant to emit VOCs that recruit egg parasitoids, as has been observed with purely phytophagous insects^4^. Heteropteran zoophytophagous predators can increase their fitness with the nutrients obtained during plant feeding by complementing or supplementing a carnivorous diet, since they can acquire from vegetable tissues not only water they need to optimize their extra-oral digestion, but also nutrients^17,52,53^. By feeding on plants zoophytophages can also induce biochemical changes in wounded plant tissue that result in production of defensive responses in host plants, similar to those produced by herbivores^23,24^. For example, the feeding and/or oviposition activities of *N. tenuis* on tomato plants activate the signalling pathway of abscisic acid that further repels the whitefly *B. tabaci*, as well as the jasmonic acid (JA) signalling pathway that serves to attract the whitefly parasitoid *E. formosa*^26,27^. Similarly, the feeding activity of two other zoophytophagous mirid species, *Macrolophus pygmaeus* (Rambur) and *Dicyphus maroccanus* Wagner, on tomato plants activates the JA signalling pathway and thereby the recruitment of *E. formosa*^25^. In our system, however, the defensive response induced in *V. faba* by feeding and oviposition activities of *P. maculiventris* affect the zoophytophagous predator directly, as the plants can recruit *T. podisi* through OIPVs emission.

In the second set of experiments, we showed that *T. podisi* females are repelled by VOCs from broad bean plants on which *H. halys*, an invasive herbivore and non-associated host, feeds and deposits eggs. Martorana *et al*.^44^ demonstrated that a local parasitoid, *T. basalis*, is not attracted by OIPVs emitted by *V. faba* plants infested by *H. halys*, suggesting that this lack of response is a consequence of the absence of coevolution among the plant, the alien herbivore and the parasitoid. For *T. basalis*, this inability to detect OIPVs to locate *H. halys* eggs could be favourable to the wasp foraging efficiency, since it prevents *T. basalis* from investing time and energy to locate poor quality host eggs, as *T. basalis* reproductive rate on *H. halys* eggs is low^44^. The inability of *T. podisi* to exploit VOCs from plants infested by *H. halys* could likewise be beneficial for this parasitoid. Indeed, native egg parasitoids frequently attack *H. halys* egg masses under field conditions, but the successful parasitism level is very low compared to those of native hosts, due to the unsuccessful development of parasitoid progeny^50^. Therefore, the invasive pest acts as an evolutionary trap and, as a consequence, local parasitoids that are not able to adapt their response to the invader could have their population levels reduced^51,54^. In our system, thus, non-attractiveness of *T. podisi* from VOCs emitted by *H. halys*-infested plants could allow the parasitoid to escape the evolutionary *H. halys* trap or limit its impact. Indeed, the lack of response by *T. podisi* to VOCs emitted by faba bean plants infested by *H. halys*, reduces the probability of *T. podisi* locating *H. halys* egg masses that are not suitable for parasitoid progeny development.

VOCs emitted by infested plants are one of the main stimuli that egg parasitoids use to locate their hosts, therefore the presence of *T. podisi* on *H. halys* egg masses observed in field conditions^55^ could be explained in terms of multi-trophic context on which other factors might affect the host location behaviour. For example, egg parasitoids can exploit volatile cues emitted by the hosts, both directly and indirectly related, and, after landing on plants, they can find the host by following chemical traces left by adult hosts^3^. Moreover, egg parasitoid attraction to OIPVs can be influenced by biotic and abiotic stress and/or by the time elapsed since the host oviposition on plant^37,38,45^.

Finally, in the last set of experiments, our behavioural bioassays showed that *T. podisi* are not attracted by *V. faba* plants concurrently infested by *P. maculiventris* and *H. halys*, The impact of invasive insect herbivores on plant volatile-mediated tritrophic signalling has been reported in a few cases and, in general, the concurrent infestation of both exotic and local insects disrupts the attraction of parasitoids towards HIPVs and OIPVs^36,56–58^. For example, Martorana *et al*.^44^ showed that *H. halys* interferes with the local established semiochemical web including *V. faba*, *N. viridula* and *T. basalis* limiting the egg parasitoid recruitment and, as a consequence, negatively affecting the egg parasitoid efficacy in controlling its associated host. The overall impact of *H. halys* on the local established semiochemical web among *V. faba*, *P. maculiventris* and *T. podisi* may have a negative effect from the parasitoid point of view, because the disruption of *T. podisi* attraction towards plants concurrently infested by the alien pest and the associated host prevent the exploitation of OIPVs to locate the suitable host. However, from an applied perspective, such a chemical disruption may have beneficial consequences to biological control. In general, plant damage caused by zoophytophagous predators is relatively low^59^, therefore, we assume that following the disruption of parasitoid foraging by *H. halys*, (1) parasitism by *T. podisi* would be reduced on *P. maculiventris*, allowing the predator to maintain its predation activity on phytophagous pests, including *H. halys*^51^; (2) the risk that *T. podisi* locates *H. halys* eggs by exploiting OIPVs from *P. maculiventris* attacked plants would be reduced, together with the risk of an ‘evolutionary trap’ for *T. podisi* progeny.

Taken as a whole, the results of our study provide new details about the response of an egg parasitoids coping with host zoophytophagous predator alone or in the presence of exotic non-host herbivore due to the reliability of OIPVs and their potential applications for ecosystem management, particularly for biological control introductions. Further studies conducted under field conditions are therefore required to better characterize multitrophic interactions involving invasive species and, forecast the efficacy of natural enemies in controlling phytophagous pests.

## Methods

### Plants

Seeds of broad bean plants (*V. faba* cv. Superaguadulce) were individually sowed in plastic pots (5 × 5 × 10 cm) filled with fertilized commercial soil (BM6, Berger, Québec, Canada). Plants were grown in a climate-controlled room (22 ± 1 °C, 50 ± 5% RH, 16 h:8 h L:D) and irrigated every two days. Plants used in the experiments were 2–3 weeks old, with approximately 7–8 fully expanded leaves.

### Insect rearing

Colonies of *H. halys* and *P. maculiventris* were established from adults collected in fields nearby Hamilton and Ottawa (Ontario, Canada) and reared separately in ventilated cages (30 × 30 × 30 cm) under controlled conditions (24 ± 2 °C; 50 ± 5% RH; 16 h:8 h L:D). The brown marmorated stink bug was fed with raw pumpkin seeds, carrots, green beans and potted soy plants. Food was renewed every two days, and water was provided with soaked cotton in small containers. The spined soldier bug was provided a diet consisting of live mealworm *Tenebrio molitor* L. (Coleoptera: Tenebrionidae) larvae, fresh green beans and *V. faba* potted plants. Egg masses were collected daily and used to maintain stink bug colonies. Stink bugs used in the experiments were from the 1^st^ to the 5^th^ laboratory generations.

The colony of *T. podisi* was established from wasps emerging from *P. maculiventris* sentinel egg masses introduced in the Montreal Botanical Garden (Québec, Canada). Wasps were reared on *P. maculiventris* egg masses, maintained in a cage (17 × 17 × 17 cm) (Bug-Dorm-44545, MegaView Science Co. Ltd., Taichung, Taiwan), fed with a honey-water solution (80:20 v/v) and kept in a controlled environment room (24 ± 1 C; 50 ± 5% RH; 16 h:8 h L:D). Daily, 10–15 egg masses were glued on paper strips and introduced into the cage. After emergence, male and female wasps were kept together to allow mating. For the experiments, wasp females, from the 1^st^ to the 7^th^ laboratory generations, 3–5 days old and naïve with respect to both oviposition experience and contact with cues released by plants and host were individually isolated in 1.2  ml Eppendorf tubes for 24 h.

### Y-tube olfactometer bioassays

The responses of *T. podisi* to plant treatments were tested in a dual-choice olfactometer consisting of a Y-shaped glass body (2 cm uniform internal diameter, 7 cm main body (stem) length, and 17 cm arm length at 165° angle). A stream of medical-grade compressed air (approximately 80:20, N2:O2) flowed through both arms. The flow was cleaned with a charcoal filter to reduce contamination from environmental cues, humidified by bubbling through a distilled water jar and regulated by flow meters to create an airstream of about 0.5 l min^−1^ per arm. The device was illuminated from above by two 18-W cool white fluorescent lamps. Before entering in the olfactometer arms, each air stream passed through a 4 l-glass jar (15 cm diameter) containing the odour sources. Tested stimuli were randomly assigned at the beginning of the bioassays and were reversed after testing three parasitoid females to avoid any bias due to eventual side preferences by the parasitoids. At every switch, the system was cleaned with fragrance-free soap, rinsed with demineralised water and dried. Wasp females were singly introduced into the Y-tube olfactometer, and their behaviour was recorded for 10 min using an HDD video camera (Sony HDR-XR500); the videos were analysed by CowLog software^60^ and parasitoid responses were measured in terms of residence time, i.e. the time spent by each wasp in the test/control arm during the bioassays. The Y-tube olfactometer bioassays were carried out as paired choices, in which the test odour sources were always tested *versus* a control odour as detailed above. Odour sources and wasp females were used only once. For each treatment, 35 replicates were conducted. The experiments were conducted from 09:00 to 14:00 in a dark room to avoid directional light, under controlled conditions (24 ± 1 °C; 50 ± 5% RH). Wasps were allowed to acclimatize for at least 1 h in the room before the experiment.

### Plant treatments

To evaluate the response of *T. podisi* females in olfactometer bioassays, we used the protocol described by Martorana *et al*.^44^. Potted broad bean plants were exposed to one stink bug female, caged for 24 h on the abaxial surface of an expanded leaf using a clip-cage, which consists of two modified plastic Petri dishes (3.5 cm diameter, 1 cm high) with a mesh-covered hole (3 cm diameter, 0.01 cm mesh) and the rim covered by a small sponge ring. Inside the clip-cage, stink bugs were allowed to feed and/or oviposit (exposed plants). Plants used for the bioassays were those on which either the stink bug feeding activity was directly observed and an egg mass was laid. Egg masses laid by *P. maculiventris* on exposed plants ranged from 13 to 25 eggs (weight average 0.056 ± 0.0011 g, N = 10,), while those laid by *H. halys* ranged from 25 to 30 eggs (weight average 0.043 ± 0.001 g, N = 10,). Treated plants with empty clip-cage, maintained on a leaf, were used as control (unexposed plants). After 24 h, stink bugs and clip-cages were removed, and 24 h later the plants were tested in the olfactometer. All the treatments were performed using from 10 to 20 days-old stink bug adult females. Parasitoid host searching behaviour was tested using odour sources combined as follow: (i) plants exposed to *P. maculiventris* feeding (Pm_F) *vs*. unexposed plants (UX); (ii) plants exposed to *P. maculiventris* feeding and oviposition (Pm_F_O) *vs*. unexposed plants (UX); (iii) plants exposed to *H. halys* feeding (Hh_F) *vs*. unexposed plants (UX); (iv) plants exposed to *H. halys* feeding and oviposition (Hh_F_O) *vs*. unexposed plants (UX); (v) plants exposed to *P. maculiventris* feeding and oviposition (Pm_F_O) and *H. halys* feeding (Hh_F) *vs*. unexposed plants (UX); (vi) plants exposed to *P. maculiventris* feeding (Pm_F) and *H. halys* feeding and oviposition (Hh_F_O) *vs*. unexposed plants (UX); (vii) plants exposed to *P. maculi*ventris feeding and oviposition (Pm_F_O) and *H. halys* (Hh_F) feeding *vs*. plants exposed to *P. maculiventris* feeding and oviposition (Pm_F_O).

### Statistical analysis

The time spent by wasp females in each arm was statistically compared by parametric paired t-tests for dependent samples. Residence time in the central arm was excluded from the analyses. Individuals that did not make a choice were excluded from the statistical analysis. Data were analysed using the STATISTICA 12 software (StatSoft, 2014).
